# An Avian Retrovirus Uses Canonical Expression and Processing Mechanisms To Generate Viral MicroRNA

**DOI:** 10.1128/JVI.02921-13

**Published:** 2014-01

**Authors:** Yongxiu Yao, Lorraine P. Smith, Venugopal Nair, Mick Watson

**Affiliations:** a Viral Oncogenesis Group, The Pirbright Institute, Compton Laboratory, Compton, Berkshire, United Kingdom; b Ark-Genomics, The Roslin Institute, R(D)SVS, University of Edinburgh, Division of Genetics and Genomics, Easter Bush, Midlothian, United Kingdom

## Abstract

To date, the vast majority of known virus-encoded microRNAs (miRNAs) are derived from polymerase II transcripts encoded by DNA viruses. A recent demonstration that the bovine leukemia virus, a retrovirus, uses RNA polymerase III to directly transcribe the pre-miRNA hairpins to generate viral miRNAs further supports the common notion that the canonical pathway of miRNA biogenesis does not exist commonly among RNA viruses. Here, we show that an exogenous virus-specific region, termed the E element or XSR, of avian leukosis virus subgroup J (ALV-J), a member of avian retrovirus, encodes a novel miRNA, designated E (XSR) miRNA, using the canonical miRNA biogenesis pathway. Detection of novel microRNA species derived from the E (XSR) element, a 148-nucleotide noncoding RNA with hairpin structure, showed that the E (XSR) element has the potential to function as a microRNA primary transcript, demonstrating a hitherto unknown function with possible roles in myeloid leukosis associated with ALV-J.

## INTRODUCTION

Retroviruses are a large group of enveloped viruses associated with a variety of diseases in a wide range of host species. Avian retroviruses, the Rous sarcoma virus (RSV) and avian leukosis virus (ALV), are historically known for their ability to induce a number of types of cancer in poultry (1). In addition to their pathogenic roles, retroviruses have provided significant insights into transcriptional regulation in a cell-type-specific manner (2). The retroviral genome includes a number of *cis*-acting elements (3). One such element is the 148-nucleotide exogenous virus-specific region (E or XSR) identified in the SR-A and Pr-C strains of RSV at the 5′ and the 3′ sides of the *src* gene, respectively (4, 5). The functions of the E (XSR) element are not clear although requirement of a 400-nucleotide region that included the E (XSR) element for oncogenicity of the recombinant avian retrovirus NTRE7 has been shown (6). The E (XSR) sequence exhibits several unusual features; it has a noncoding RNA capable of forming characteristic hairpin structures (7). From its location at two different sites on either side of the *src* gene in the two RSV strains, it is clear that the functions of E (XSR) can be exerted over distance. Based on these observations, it was speculated that E (XSR) may function as a transcriptional enhancer (5).

Interest in the E (XSR) element was revived when it was demonstrated in the 3′ noncoding region of the genome of HPRS-103, the ALV subgroup J (ALV-J) prototype virus (8), identified in the United Kingdom in 1988 as the causative agent of myeloid leukosis, which rapidly became a worldwide health and welfare problem in chickens (9–13). The E (XSR) sequence is conserved in the majority of the ALV-J isolates although deletions or modifications in this sequence have also been seen (13–16). The role of the E (XSR) element in the pathobiology of ALV-J is not known although potential C/EBP and c-Ets-1 binding sites have been predicted in the sequence (13, 14). However, ALV-J strains with deletions or mutations in the E (XSR) element have also been isolated from clinical cases (9–11, 14, 17). Our previous studies using HPRS-103 clones with precise deletions in the E (XSR) element indicated that these elements are essential for oncogenicity, but this was related to the genetic background of the birds (7). Despite the presence of the E (XSR) element and its association with the oncogenicity of RSV and ALV-J, the molecular mechanisms of the E (XSR) element functions remain unclear. Although an enhancer-like function has been speculated (5, 14), firm supporting evidence is still lacking.

In many organisms, including several viruses, microRNAs (miRNAs) are well recognized as major regulators of gene expression (18). Given their profound ability to regulate multiple targets, these molecules are exploited particularly by several DNA viruses as tools for manipulating the cellular environment (19, 20). RNA viruses are generally thought not to contain pre-miRNA structures to avoid endonuclease-mediated cleavage of the genome, antigenome, and mRNAs. Although retroviruses have not been widely documented to exploit the miRNA pathway (21), a recent demonstration of a conserved cluster of RNA polymerase III (Pol III)-transcribed miRNAs from the bovine leukemia virus (BLV) genome (22, 23) showed the potential of retroviruses to encode miRNAs. The E (XSR) element sequences from ALV-J strains show hairpin-like structures suggestive of miRNA precursors although the existence of any mature miRNA has not been demonstrated in ALV-J-infected/transformed cells. Using a deep-sequencing approach on one of the ALV-J-transformed cell lines, we identified a novel small-RNA population encoded from within the E (XSR) element.

## MATERIALS AND METHODS

### Cells.

HEK293T cells and the chicken embryo fibroblast (CEF) cell line DF-1 (24) were maintained in Dulbecco's modified Eagle's medium (DMEM) supplemented with 10% fetal calf serum (FCS) (Sigma). A reticuloendotheliosis virus T (REV-T)-transformed turkey spleen cell (TSC) line, AVOL-1T, and IAH30, a turkey macrophage (MΦ) cell line (25) transformed by the acutely transforming ALV subgroup J 966 virus with a transduced v-*myc* oncogene (26), were grown at 38.5°C in 5% CO_2_ in RPMI 1640 medium containing 10% FCS, 2% chicken serum, 10% tryptose phosphate broth, 0.1% 2-mercaptoethanol, and 1% sodium pyruvate. AVO4-1B3 cells, an avian blastoderm cell line transformed by acutely transforming ALV-J isolate 1B (27), was maintained in Eagle's minimal essential medium (EMEM) supplemented with 10% FCS. Turkey spleen cells were prepared from spleen tissues of uninfected turkey by using Histopaque-1083 (Sigma-Aldrich) density gradient centrifugation. Chicken macrophages were prepared from bone marrow of adult uninfected chickens using procedures described previously (12). Briefly, femoral bone marrow was flushed out with RPMI medium, and the contents were passed through a cell sieve. After Histopaque-1083 density gradient centrifugation, the cells from the interface were collected, washed twice using RPMI medium supplemented with 5% FCS and 5% chicken serum, plated into 60-mm dishes, and cultured at 38.5°C in 5% CO_2_. The medium was changed after 3 days, and the cells were used for virus infection.

### Plasmids.

The miR-155 expression plasmid pEF6-Bic was described previously (28). The MHV-miR-M1-7-3p expression plasmid pIDTSmartKan-MHV68-M1-7 was kindly provided by Chris Sullivan, University of Texas, Austin, TX. The E (XSR) miRNA expression cassette was PCR amplified from HPRS-103 genomic DNA with primers 5′-TCGATAGGAAGCTTAAAGCAGTGCATGGGTAGGGGT-3′ and 5′-TCACGTAATCTAGACCACCTTACTTCCACCAATCGACG-3′. The isolated fragments were either cloned into pGEM-T-easy by TA cloning or into pcDNA3.1-His/myc via restriction enzyme sites HindIII and XbaI. A luciferase sensor reporter was constructed by synthesizing four tandem repeats of artificial target sites with a perfect match to E (XSR) miRNA as sense and antisense oligomers; the construct was annealed and cloned into the 3′ untranslated region (UTR) of the Renilla luciferase gene through the NotI-XhoI site in the psiCHECK-2 vector (Promega). A control mutant reporter construct (sensor-mu) was generated alongside the wild-type reporter construct to be the negative control. In the mutant construct, nucleotides 2, 4, 6, and 8 of the microRNA response element (MRE) were changed to different bases, thus preventing recognition of the target site by the miRNA. In all cases, the cloned sequences were confirmed by sequence analysis.

### Deep sequencing of an IAH30 small-RNA library.

RNA extraction for miRNA profiling was carried out as previously described (29) using an miRVana miRNA isolation kit (Ambion, United Kingdom). Sequencing of the miRNAs was carried out on an Illumina GAIIx platform by GATC Biotech. After sequencing, adaptor and primer/dimer sequences were removed, yielding approximately 1.5 million 36-bp single-end reads. Using the Novoalign short-read mapper (Novocraft, Selangor, Malaysia), we mapped the reads to the known chicken mature miRNAs downloaded from miRBase (www.miRBase.org) and the HPRS-103 genome sequence (GenBank accession number Z46390.1). To correct for differences in library size and sequencing depth, raw mapped-read counts were scaled to reads per kilobase per million sequenced reads (30).

### Northern blotting.

Total RNA was extracted from cultured cells with an miRNeasy Kit (Qiagen) according to the manufacturer's instructions. Samples of 20 μg of total RNA were resolved using a 15% polyacrylamide–1× Tris-borate–EDTA–8 M urea gel and blotted to a GeneScreen Plus membrane (Perkin-Elmer). The probe sequences of DNA oligonucleotides with sequences complementary to candidate miRNAs are as follows: miR-155 probe, 5′-CCCCTATCACGATTAGCATTAA-3′; E (XSR) miRNA probe, 5′-CAGAGGCAACTTGAATAGTCTA-3′; and MHV68-M1-7 probe, 5′-AATAAAGGTGGGCGCGATATC-3′. The 5.8S probe sequence is 5′-TTCTTCATCGACGCACGAGC-3′. The probes were end labeled with [γ-^32^P]ATP (Amersham) and T4 polynucleotide kinase (New England BioLabs) to generate high-specific-activity probes. Hybridization, washing, and autoradiography were carried out as previously described (31).

### Dual-luciferase assay.

The transfection of DF-1 cells and Vero cells was carried out with Lipofectamine 2000 (Invitrogen). CEFs were transfected with Lipofectamine (Invitrogen), and IAH30 cells were transfected with a Nucleofactor Transfection Kit T (Lonza). E (XSR) miRNA mimics were synthesized by Qiagen. Approximately 3 × 10^4^ cells were seeded in each well of a 96-well plate. Except for the IAH30 cell line, which was transfected with reporter construct alone, DF-1, CEF, and Vero cells were cotransfected with 20 ng of each reporter construct in psiCHECK-2 vector along with either 100 ng of (E) XSR miRNA expression plasmid (DF-1) or a final concentration of 20 nM E (XSR) miRNA mimics (DF-1, CEF, and Vero cells) using different transfection reagents, as stated above, as per the manufacturer's protocols. In all cases, constitutively expressed firefly luciferase activity in the psiCHECK-2 vector served as a normalization control for transfection efficiency.

### RNA polymerase II dependence assay.

HEK293T cells in six-well plates were transfected with 2.5 μg of miRNA expression vector using Lipofectamine 2000 and, where indicated, were then treated 2 h later with a final concentration of 50 μg/ml α-amanitin (32). Total RNA was extracted at 24 h posttransfection, and Northern blot analysis was performed.

### RNA interference (RNAi) assays.

Silencer Select validated siRNAs against human Drosha (siRNA identification numbers [ID], s26491 and s26492, referred to as D1 and D2) and Dicer (siRNA ID s23754) were purchased from Ambion. They have been verified experimentally by the company in cell-based assays to reduce the expression of their individual target genes by 80% in at least three biological replicates. HEK293T cells in six-well plates were transfected with 20 nM Drosha or Dicer siRNA using Lipofectamine RNAi-MAX (Invitrogen) following the manufacturer's recommendations. At 24 h posttransfection, cells were cotransfected with 20 nM each siRNA and 2 μg of miRNA expression plasmid using Lipofectamine 2000. Twenty-four hours later, RNA was extracted, and Northern blot analysis was performed.

### Stem-loop quantitative reverse transcription-PCR (qRT-PCR) for E (XSR) miRNA.

Total RNA was extracted from cultured cells with an miRNeasy Kit (Qiagen) according to the manufacturer's instructions. miRNAs were quantified using custom TaqMan stem-loop microRNA assays (ABI) according to the manufacturer's recommendations using 10 ng of total RNA as a template for reverse transcription with the primer 5′-GTCGTATCCAGTGCAGGGTCCGAGGTATTCGCACTGGATACGACCAGAGG-3′, followed by quantitative PCR carried out using the forward primer 5′-CGGTCGACCTAGACTATTCAAGTTG-3′, reverse primer 5′-CAGTGCAGGGTCCGAGGT-3′, and probe 5′-TGGATACGACCAGAGGC-3′. A TaqMan microRNA assay for let-7a (assay ID 000377) was purchased from ABI. Each reverse transcription reaction was performed twice independently, and each reaction mixture was used for triplicate PCR. The level of miRNA expression is presented as fold expression relative to the background amplification obtained with RNA isolated from either chicken MΦ (see Fig. 2B) or untransfected DF-1 (see Fig. 4A) and after normalization to the ubiquitously expressed cellular miRNA let-7a.

## RESULTS

### E (XSR) element encodes a novel miRNA.

**FIG 1:**
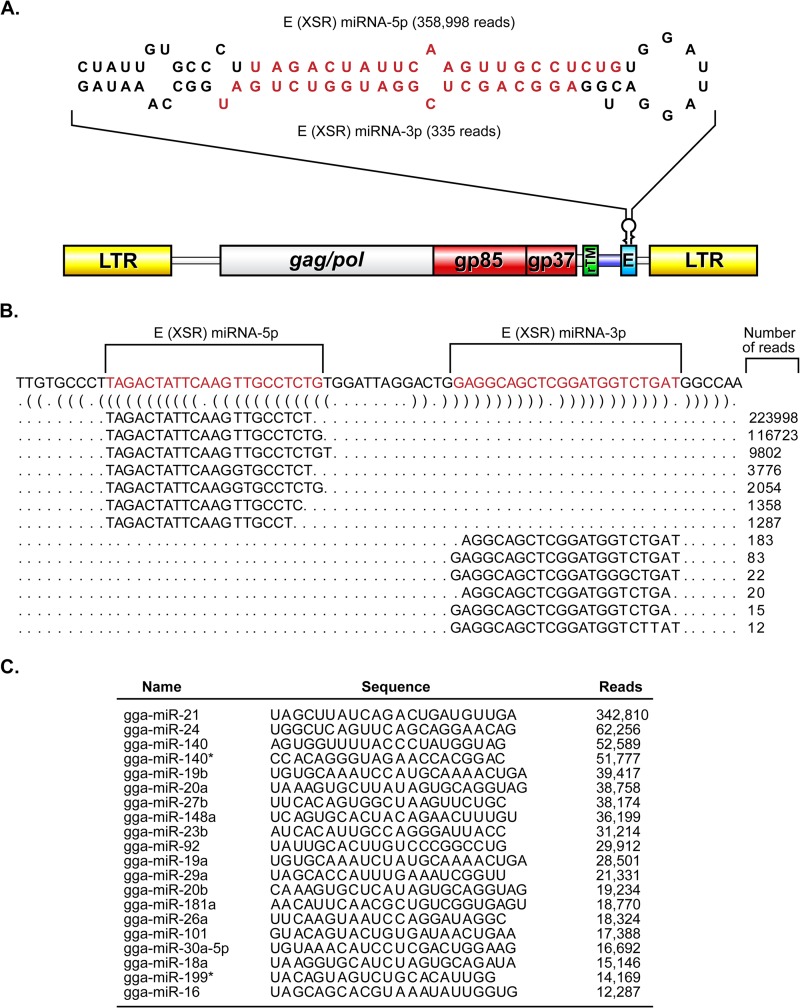
The E (XSR) element in the ALV-J genome encodes a novel microRNA. (A) Genomic location of E (XSR) miRNA. The structure of the ALV-J provirus HPRS-103 genome with the long terminal repeat (LTR), *gag*/*pol*, *env* (gp85 and gp37), redundant transmembrane (rTM), and the E element is shown. The secondary structure of E (XSR) pre-miRNA predicted using the MFOLD algorithm is shown on the top. The mature miRNA strands are indicated in red. The mature miRNAs with the number of reads for each strand recovered from deep sequencing are indicated. (B) Reads representing the major populations of E (XSR) miRNA-5p and E (XSR) miRNA-3p aligned against the precursor sequence. Dot-bracket notation was produced by applying RNAfold. (C) Most-often-sequenced small RNA sequences from IAH30 that map to Gallus gallus miRNAs (gga prefix) listed in miRBase.

During miRNA profiling of an ALV-J-transformed turkey macrophage cell line IAH30 (25) by deep-sequence analysis on an Illumina GAIIx platform, we observed the presence of two distinct small RNA sequences that mapped perfectly to the E (XSR) element in the 3′ UTR of the HPRS-103 genome (GenBank accession number Z46390.1). The total reads of the two small RNA sequences account for 24.5% of the total IAH30 “miRNAome.” Notably, only 580 reads were scattered across the 7,841-bp genome outside this region. The relative abundance (358,998 and 335 reads, respectively) (Fig. 1A and B) and size of these small RNAs suggested they are miRNA candidates. Closer examination of the sequence revealed that it is derived from the hairpin structure of the E (XSR) element (Fig. 1A). During miRNA biogenesis, processing of the pre-miRNA yields the more abundant miRNA and less abundant passenger strands (33). From our data, we conclude that the abundant species that mapped to 5′ arm of the stem-loop sequence is likely to be the miRNA strand, while the less abundant sequence that mapped to 3′ arm of the stem-loop represented the passenger strand. Importantly, the 5′ arm of the stem-loop sequence is the most abundant species present in the IAH30 library (Fig. 1C). Taken together, these results strongly suggested that we had identified a pre-miRNA encoded within the E (XSR) element of ALV-J.

**FIG 2:**
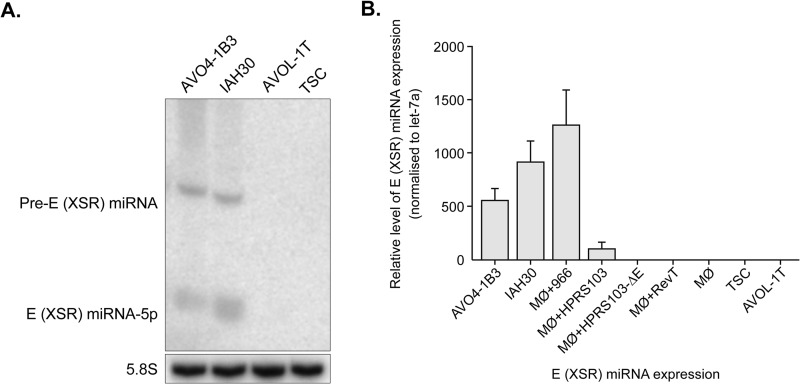
E (XSR) miRNA is expressed in ALV-J-transformed cells. (A) Northern blot analysis of RNA from the blastoderm cell line AVO4-1B3, turkey macrophage cell line IAH30, REV-T-transformed turkey spleen cell line AVOL-1T, and turkey spleen cells (TSCs) demonstrating the expression of mature and pre-miRNA of E (XSR) miRNA. The cellular 5.8S RNA served as the loading control. (B) E (XSR) miRNA expression levels determined by qRT-PCR. Relative expression of E (XSR) miRNA measured in RNA extracted from AVO4-1B3, IAH30, and chicken macrophages infected with either 966 (MΦ+966), HPRS-103 (MΦ+HPRS103), HPRS-103 with a deletion of the E element (MΦ+HPRS103-ΔE), or REV-T (MΦ+RevT) as well as AVOL-1T cells and TSCs compared to uninfected macrophage (MΦ). Results represent the means of triplicate assays with error bars showing the standard error of the means.

In order to confirm that these novel miRNAs identified in the IAH30 cell line were indeed derived from the E (XSR) sequences in the HPRS-103 genome and did not originate from the turkey cells, we carried out Northern blotting hybridization with the miRNA strand probe. Northern blotting detected both mature miRNA and pre-miRNA in IAH30 cells. No miRNAs were detected with RNA extracted from the reticuloendotheliosis virus T (REV-T)-transformed turkey cell line AVOL-1T and uninfected turkey spleen cells (TSCs) (Fig. 2A). This finding was further confirmed by TaqMan miRNA assays (Fig. 2B). IAH30 is a turkey macrophage cell line transformed by strain 966, an acutely transforming virus derived from HPRS-103. To test that the expression of the E (XSR)-derived miRNA is not limited to the transformed macrophages of turkey origin, we also examined the primary chicken macrophages infected with either HPRS-103 or 966 virus. RNA isolated from the infected cells was tested for E (XSR) miRNA expression using a TaqMan assay. Indeed, E (XSR) miRNA was detected in cells infected with both types of viruses (Fig. 2B). Furthermore, E (XSR) miRNA was also detected in AVO4-1B3 cells, a chicken blastoderm cell line transformed by another acutely transforming ALV-J isolate, 1B (27), by both Northern blotting and TaqMan miRNA assay (Fig. 2A and B). The E (XSR) miRNA was not detected with RNA extracted from macrophages infected with HPRS-103 with a deletion of E (XSR) (7) or from uninfected or REV-T-infected macrophages (Fig. 2 B). The fact that E (XSR) miRNA was expressed in different cell types infected with a number of different ALV-J virus strains further confirmed that E (XSR) miRNA is indeed a genuine miRNA encoded by ALV-J virus.

### E (XSR) miRNA sequence is highly conserved.

We next examined the evolutionary conservation of E (XSR) miRNA by aligning all pre-miRNA sequences of NCBI-deposited ALV subgroup A, subgroup J, and Rous sarcoma virus isolates with E element sequences along with the pre-miRNA sequence of E (XSR) miRNA from HPRS-103 (see Fig. S1 in the supplemental material). Within the 64 aligned pre-miRNA sequences of the E (XSR) element, the miRNA strand (5′ arm, miRNA-5p) was identical in 46 isolates, with 16 isolates showing a single nucleotide change and the remaining 2 isolates showing 2-nucleotide differences. The seed region showed only a single nucleotide substitution among all the sequences, suggesting evolutionary pressure on maintaining the sequence of this region, potentially for modulating the expression of its targets. The sequence of the passenger strand (3′ arm, miRNA-3p), on the other hand, showed more substitutions. The loop region was highly conserved, and despite deletions or insertions in this region, all of the pre-miRNA sequences were able to form hairpin structures (data not shown). The fact that the miRNA sequence is well conserved across all known ALV-J isolates with an E (XSR) element suggests that this newly identified ALV-J miRNA may have a conserved functional role.

### E (XSR) miRNA is processed by the canonical miRNA biogenesis pathway.

**FIG 3:**
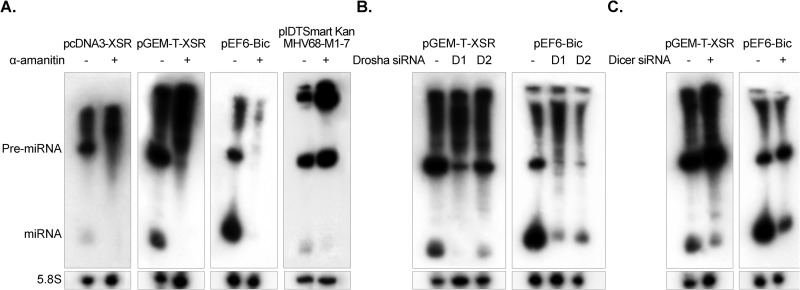
E (XSR) miRNA is processed by the canonical miRNA biogenesis pathway. (A) Northern blot analysis of E (XSR) miRNA expression plasmids pcDNA3-XSR and pGEM-T-XSR, plasmid pEF6-Bic expressing Gallus gallus miR-155 (gga-miR-155), and MHV68-miR-M1-7 expression plasmid pIDTSmart-Kan-MHV68-miR-M1-7 transfected into HEK293T cells with or without treatment of the Pol II inhibitor α-amanitin. The cellular 5.8S RNA was used as a loading control. (B) Northern blot analysis of E (XSR) miRNA and gga-miR-155 expression vectors transfected into HEK293T cells with or without siRNA against human Drosha (D1 and D2). 5.8S RNA was used as a loading control. (C) Northern blot analysis of E (XSR) miRNA and gga-miR-155 expression vectors transfected into HEK293T cells with or without siRNA against human Dicer. 5.8S RNA was used as a loading control.

The vast majority of viral miRNAs are transcribed by RNA polymerase II (Pol II) before being processed by RNase III enzymes Drosha and Dicer. The exceptions are the Pol III-derived tRNA-like precursor structures of mouse hepatitis virus 68 (MHV68) (32, 34) and the recently reported BLV miRNAs (22, 23, 35). In the absence of detecting any sequence motifs of Pol III promoter elements (22, 23) in the E (XSR) flanking sequence, we hypothesized that this miRNA is Pol II derived. To test this, we cloned the stem-loop structure sequence together with approximately 150 nucleotides of flanking sequence downstream of the cytomegalovirus (CMV) promoter into pcDNA3 vector (Invitrogen). High-level expression of E (XSR) miRNA detected by Northern blotting in transfected HEK293T cells suggested that the E (XSR) miRNA is processed by the Pol II promoter (Fig. 3A). The hypothesis of Pol II driving expression was further explored by testing the blockage of miRNA production by treatment with α-amanitin, a selective inhibitor of Pol II (22, 32). We transfected HEK293T cells with the plasmid expressing E (XSR) miRNA in pcDNA3 or pGEM-T easy vector in the presence or absence of α-amanitin. As shown in Fig. 3A, α-amanitin inhibits both pre-miRNA and mature miRNA of the E (XSR) miRNA, thus supporting our initial Pol II prediction. A cellular miRNA miR-155 transcribed under the control of the Pol II promoter pEF6-Bic and a virus miRNA, MHV68-miR-M1-7-3p (kindly provided by C. Sullivan), transcribed under the control of a Pol III promoter were used as controls. As expected, expression of both E (XSR) miRNA and miR-155 was blocked by α-amanitin treatment, whereas the MHV68-miR-M1-7-3p miRNA was resistant (Fig. 3A). This further confirms that the expression of E (XSR) miRNA is driven by Pol II promoter but not Pol III promoter.

Drosha is a key enzyme in microRNA biogenesis, generating the pre-miRNA by excising most pre-miRNA structures from Pol II transcripts (36). To determine whether Drosha contributes to E (XSR) miRNA processing, we used RNA interference (RNAi) to knock down Drosha in HEK293T cells. Two Silencer Select validated siRNAs against human Drosha (Life Technologies) were used for the knockdown of Drosha expression. We cotransfected HEK293T cells with siRNA against Drosha and vectors expressing either E (XSR) miRNA or control miRNA miR-155 (Fig. 3B). Knockdown of Drosha by either siRNA resulted in a marked decrease in the miR-155 expression which is known to be Drosha dependent (Fig. 3B). Similar results were obtained with E (XSR) miRNA expression following Drosha knockdown, suggesting that E (XSR) miRNA expression is also Drosha dependent.

Dicer is the second RNA III enzyme in the miRNA biogenesis pathway responsible for cleavage of pre-miRNA to generate mature miRNA. To confirm the contribution of Dicer to E (XSR) miRNA expression, we cotransfected HEK293T cells with validated siRNA against Dicer (Life Technologies) and vectors expressing either E (XSR) miRNA or control miRNA miR-155 (Fig. 3C). As expected, knockdown of Dicer resulted in an increase of the ratio of pre-miRNA to miRNA for both E (XSR) miRNA and Dicer-dependent miR-155 (Fig. 3C). Thus, we conclude that E (XSR) miRNA is processed by the canonical miRNA biogenesis pathway.

### E (XSR) miRNA is a biologically functional miRNA.

**FIG 4:**
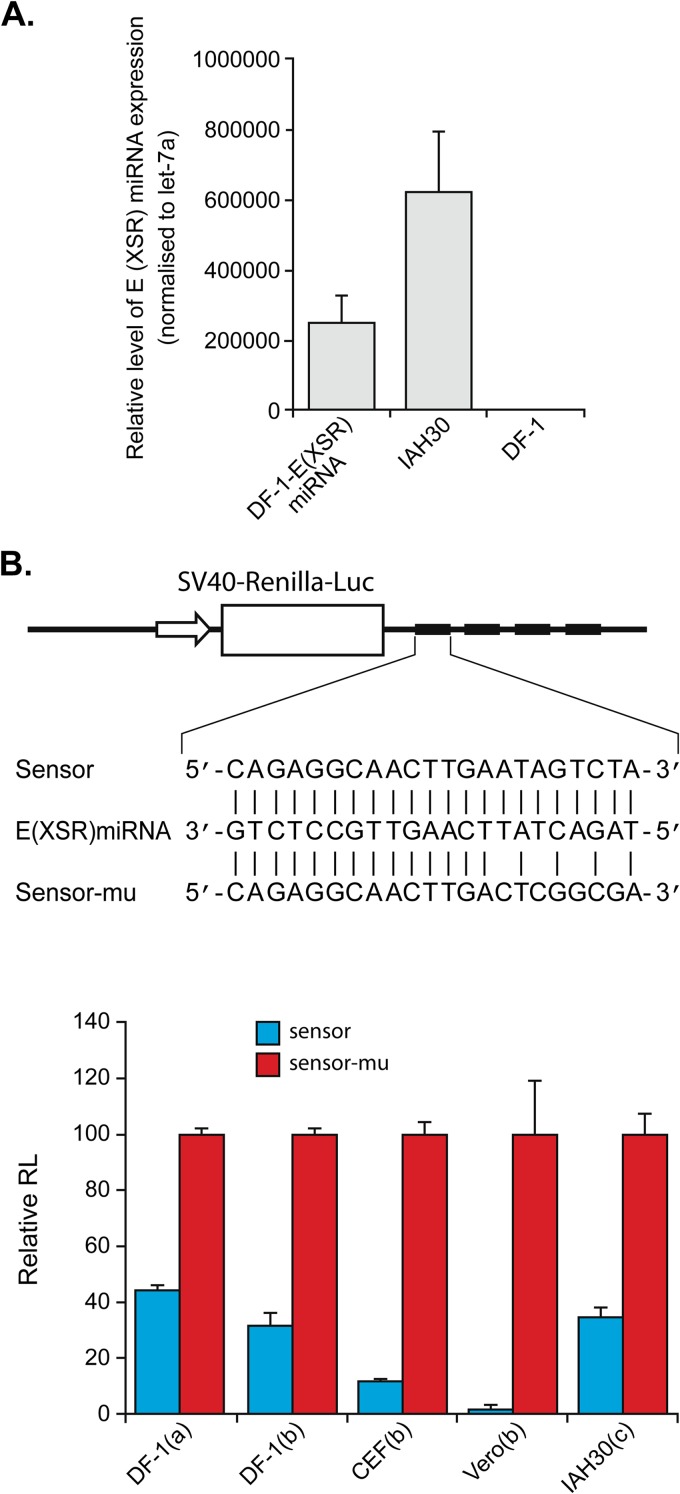
E (XSR) miRNA is a biologically functional miRNA. (A) E (XSR) miRNA expression levels determined by qRT-PCR. Relative expression of E (XSR) miRNA measured in RNA extracted from miRNA expression plasmid-transfected DF-1 and IAH30 cells compared to untransfected DF-1 cells. Results represent the means of triplicate assays, with error bars showing the standard errors of the means. (B) The top panel shows a sensor construct with four tandem repeats of artificial target sites of perfect complementarity to the miRNA sequence inserted into the 3′ UTR at the end of the Renilla luciferase gene in psiCHECK-2 vector (sensor) and a sensor mutant (sensor-mu) construct with four mutated nucleotides in the seed region. The bottom panel shows repression of luciferase by the sensor construct relative to the mutant construct after cotransfection with an E (XSR) miRNA expression plasmid into DF-1 cells (a) or cotransfection with E (XSR) miRNA mimics into DF-1, CEF, and Vero cells (b) and transfection into IAH30 (c). The relative expression of Renilla luciferase (RL) was determined with the normalized levels of firefly luciferase. For each sample, values from four replicates representative of at least two independent experiments were used in the analysis. SV40, simian virus 40.

To determine whether the E (XSR) miRNA is biologically functional, psiCHECK-2 luciferase-based reporter plasmids, bearing four tandem repeats of artificial target sites of perfect complementarity to the miRNA sequence inserted into the 3′ UTR (Fig. 4B), either were cotransfected into DF-1 cells along with the E (XSR) miRNA expression plasmid or along with the E (XSR) miRNA mimics (Qiagen) into either DF-1 cells and Vero cells using Lipofectamine 2000 (Invitrogen) or chicken embryo fibroblasts (CEF) using Lipofectamine (Invitrogen), or they were transfected into IAH30 using Nucleofactor Transfection Kit T (Lonza). The expression of E (XSR) miRNA from transfected DF-1 and in IAH30 cells was confirmed by quantitative TaqMan miRNA assay (Fig. 4A). As shown in Fig. 4B, E (XSR) miRNA expressed from an expression plasmid was able to inhibit luciferase reporter expression by 56% in DF-1 cells relative to expression from a mutant reporter plasmid with four mutated nucleotides in the seed region. E (XSR) miRNA mimics reduced the luciferase level by 68% in DF-1 cells, 88% in CEFs, and 98% in Vero cells, and the endogenous E (XSR) miRNA in IAH30 cells could inhibit luciferase expression by 65%. Thus, the reporter assay demonstrated that the miRNA is active within the RNA-induced silencing complex (RISC) and that the E (XSR) element is processed into functional mature miRNA in DF-1 cells.

## DISCUSSION

Of the 295 virus-encoded miRNAs deposited in the miRBase database, the vast majority are encoded by DNA viruses. The small size and lack of immunogenicity, combined with the ability for specific repression of the expression of multiple target transcripts, make the miRNAs ideal tools for the viruses to reshape gene expression in an infected cell to favor viral replication and pathogenesis. Although there is a long way to go to gain significant understanding of how these miRNAs function and of the portfolio of their targets, it is clear that these small but effective regulators of gene expression play a key role in virus biology.

Among all viruses, the members of the family *Herpesviridae* account for the majority of the currently known virus-encoded miRNAs (19, 37). In addition to the herpesviruses, a small number of other nuclear DNA viruses, particularly polyomaviruses, have also been shown to encode miRNAs or miRNA-like molecules (38–45). Furthermore, no viral miRNAs have been identified using low- or high-throughput sequencing of RNA from cultured cells infected with any of several different RNA viruses, including hepatitis C virus (HCV), yellow fever virus (YFV), West Nile virus (WNV), human papillomavirus (HPV), vesicular stomatitis virus (VSV), dengue virus, polio virus, human T-cell leukemia virus type 1 (HTLV-1), and influenza A virus (21, 34, 46–48). Although there have been several reports suggesting that HIV, an RNA virus whose genome is reverse transcribed and incorporated into the host DNA, may also encode miRNAs, these reports are controversial (21, 34). Thus, a widely accepted example of any naturally RNA virus-encoded miRNA was lacking until the recent report of miRNAs encoded by bovine leukemia virus (BLV), a retrovirus with an RNA genome (22, 23). The most compelling hypothesis for the lack of miRNA sequences in the RNA virus genome is that excision of an miRNA from an RNA virus would result in the cleavage and ultimately the destruction of the viral genomic RNA, which would likely inhibit virus replication. This hypothesis is supported by the finding that BLV can overcome this obstacle by encoding pre-miRNA structures that are only competent to be processed into miRNAs when they are generated from subgenomic Pol III-derived transcripts (22, 23).

In spite of this theoretical barrier, here we provide evidence that ALV-J uses Pol II for the production of high-level E (XSR) miRNA. Subsequently, we have shown that the processing of the E (XSR) miRNA is Drosha and Dicer dependent. Taken together, this is the first example of an RNA virus that encodes an miRNA using the canonical miRNA biogenesis pathway. This suggests that the RNA viruses could utilize different strategies to express their own miRNAs and that they could tolerate *cis* cleavage within the genome during pre-miRNA processing. Indeed, retroviruses, a flavivirus, and influenza virus can be engineered to express biologically active miRNAs or miRNA-like molecules (49–51). The evidence of BLV miRNAs transcribed by the Pol III promoter and ALV E (XSR) miRNA transcribed by the Pol II promoter suggests that future miRNA discovery efforts could be directed to other retroviruses.

One of the conspicuous findings from the analysis of the miRNA sequences of the IAH30 library was that E (XSR) miRNA accounted for a quarter of the 1.469 × 10^6^ sequences of the IAH30 miRNAome. An increased proportion of virus-encoded miRNAs to host-encoded miRNAs is not uncommon in transformed cell lines. For example, miRNAs encoded by Kaposi's sarcoma-associated herpesvirus and Epstein-Barr virus (EBV) accounted for ∼40% of the entire miRNA pool identified from the BC-1 cell line coinfected with these two viruses (52). The total proportion of virus-encoded miRNAs of Marek's disease virus type 1 (MDV-1) and Marek's disease virus type 2 (MDV-2) in an MSB-1 cell library was 61% (53). The high-level expression of viral miRNAs has been linked to their role in virus-induced oncogenesis since the cluster 1 miRNAs of MDV-1 and mdv1-mir-M4-5p, a member of cluster 1 miRNA and a functional ortholog of gga-mir-155 which are highly expressed in the transformed cell lines and in tumors, have been shown to play a key role in MDV-1-induced tumorigenesis (54). A high level of expression of E (XSR) miRNA in the IAH30 cell line suggests that this miRNA has a major role in ALV-J pathogenesis and neoplastic transformation. Although the E element *per se* is not absolutely essential for tumor induction by this subgroup of viruses, our previous work comparing the oncogenicity of viruses derived from the parental HPRS-103 virus and from HPRS-103 with a deletion of the E element in two genetically distinct lines of birds showed that the E element does contribute to oncogenicity in certain genetic lines of chicken (7). Future studies comparing the genomes of these lines could provide insights into the polymorphisms, including those in any potential E (XSR) miRNA target sites that could account for such differential susceptibility phenotypes among these lines. The discoveries of the virus-encoded miRNA targets would help us to get a clearer understanding of the role played by the viral miRNAs.

In summary, we demonstrated that an RNA virus expresses abundant, evolutionarily conserved miRNA using the canonical miRNA biogenesis pathway. The identification of this novel potentially functional miRNA adds yet another regulatory mechanism in the pathobiology of ALV and RSV.

## Supplementary Material

Supplemental material
