# Supplementary material for: An Avian Retrovirus Uses Canonical Expression and Processing Mechanisms To Generate Viral MicroRNA
Source: J Virol. 2014 Jan;88(1):2–9. doi: 10.1128/JVI.02921-13 (PMC3911700; doi:10.1128/JVI.02921-13)
Supplement: Supplemental material [file supp_88_1_2__index.html]

An Avian Retrovirus Uses Canonical Expression and Processing Mechanisms To Generate Viral MicroRNA — Supplemental material 

# An Avian Retrovirus Uses Canonical Expression and Processing Mechanisms To Generate Viral MicroRNA

## Supplemental material

**Files in this Data Supplement:**

- Supplemental file 1 -

  Fig. S1 (Alignment of the microRNA sequence.)

  PDF, 2.5M
