# Supplementary material for: An Avian Retrovirus Uses Canonical Expression and Processing Mechanisms To Generate Viral MicroRNA
Source: J Virol. 2014 Jan;88(1):2–9. doi: 10.1128/JVI.02921-13 (PMC3911700; doi:10.1128/JVI.02921-13)
Supplement: Supplemental material [file JVI.02921-13_zjv999098470so1.pdf]

Fig. S1 E (XSR) miRNA sequences are conserved

```
                                     E (XSR) miRNA-5p         loop region          E (XSR) miRNA-3p
Z46390.1|    --------------TGCCCTTAGACTATTCAAGTTGCCTCTGTGG-----------ATTA--GGACTGGAGGCAGCTCGGATGGTCTGATGGCCA-- 68
HM452342.1|  ----------GTTTTGCCCTTGGACTATTCAAGTTGCCTCTGTGA-----------ATTG--GGGCTGGAGGCAGCTCGGATGGTCTGCTGGCCA-- 78
AY689440.1|  ----------GTTTTGTCCTTAGACTATTCAAGTTGCCTCTGTGG-----------A--------CTGGAGGCAGCTCGGATGGTCTGATGGCCA-- 66
L29199.1     ----------GTTTTGCCCTTAGACTATTCAAGTTGCCTCTGTGG-----------ATTA--GGGCTGGAGGCAGCTCGGATGGTCTGACGGCCAGA 74
V01169.1|    ----------GTTTTGCCCTTAGACTATTCAAGTTGCCTCTGTGG-----------ATTA--GGGCTGGAGGCAGCTCGGATGGTCTGACGGCCAGA 74
D10652.1     ----------GTTTTGCCCTTAGACTATTCAAGTTGCCTCTGTGG-----------ATTA--GGGCTGGAGACAGCTCGGATGGTCTGACGGCCAGA 74
U41727.1|    ----------GTTTTGCCCTTAGACTATTCAAGTTGCCTCTGTGG-----------ATTA--GGGCTGGAGGCAGCTCGGATGGTCGGACGGCCAGA 74
AF052428.1|  -------- -GTTTTGCCCTTAGACTATTCAAGTTGCCTCTGTGG-----------ATTA--GGGCTGGAGGCAGCTCGGATGGTCGGACGGCCAGA 80
K00928.1|    ----------GTTTTGCCCTTAGACTATTCAAGTTGCCTCTGTGG-----------ATTA--GGGCTGGAGGCAGCTCGGATGGTCGGACGGCCAGA 74
DQ365814.1   ----------GTTTTGCCCTTAGACTATTCAAGTTGCCTCTGTGG-----------ATTA--GGGCTGGAGACAGCTCGGATGGTCTGATGGCCAGA 74
HM776937.1   ----------GTTTTGCCCTTAGACTATTCAAGTTGCCTCTGTGG-----------ATTA--GGGCTGGAGGCAGCTCGGATGGTCTGATGGCCAGA 74
M30022.1|    ----------GTTTTGCCCTTAGACTATTCAAGTTGCCTCTGTAG-----------ATTA--GGGCTGGAGGCAGCTCGGATGGTCTGATGGCCAGA 74
X12707.1|    ----------GTTTTGCCCTTAGACTATTCAAGTTGCCTCTGTGG-----------ATTA--GGGCTGGAGGCAGCTCGGATGGTCTGATGG----- 69
X06198.1|    ----------GTTTTGCCCTTAGACTATTCAAGTTGCCTCTGTGG-----------ATTA--GGGCTGGAGGCAGCTCGGATGGTCTGATGG----- 69
X15345.1|    ----------GTTTTGCCCTTAGACTATTCAAGTTGCCTCTGTGG-----------ATTA--GGGCTGGAGGCAGCACGGATAGTCTGATGGCCA-- 78
X52822.1|    ----------GTTTTGCCCTTAGACTATTCAAGTTGCCTCTGTGG-----------ATTA--GGGCTGGAGGCAGCACGGATAGTCTGATGGCCA-- 72
M14897.1|    ----------GTTTTGCCCTTAGACTATTCAAGTTGCCTCTGTGG-----------ATTA--GGGCTGGAGGCAGCACGGATAGTCTGATGGCCA-- 72
J02342.1|    ----------GTTTTGCCCTTAGACTATTCGAGTTGCCTCTGTGG-----------ATTA--GGGCTGGAGGCAGCACGGATAGTCTGATGGCCA-- 78
M21955.1|    ----------GTTTTGCCCTTAGACTATTCGAGTTGCCTCTGTGG-----------ATTA--GGGCTGGAGGCAGCACGGATAGTCTGATGGCCA-- 72
M16582.1|    ----------GTTTTGCCCTTAGACTATTCGAGTTGCCTCTGTGG-----------ATTA--GGGCTGGAGGCAGCACGGATAGTCTGATGGCCA-- 72
AF033808.1|  ----------GTTTTGCCCTTAGACTATTCGAGTTGCCTCTGTGG-----------ATTA--GGGCTGGAGGCAGCACGGATAGTCTGATGGCCA-- 78
V01197.1|    ----------GTTTTGCCCTTAGACTATTCGAGTTGCCTCTGTGG-----------ATTA--GGGCTGGAGGCAGCACGGATAGTCTGATGGCCA-- 72
X13818.1|    ----------GTTTTGCCCTTAGACTATTCGGGTTGCCTCTGTGG-----------ATTA--GGGCTGGAGGCAGCACGGATAGTCTGATGGCCA-- 72
X51863.1|    ----------GTTTTGCCCTTAGACTATTCAGGTTGCCTCTGTGG-----------ATTA--GGGCTGGAGGCAGCACGGATAGTCTGATGGCCA-- 72
X68524.1|    ----------GTTTTGCCCTTAGACTATTCAGGTTGCCTCTGTGG-----------ATTA--GGGCTGGAGGCAGCACGGATAGTCTGATGGCCA-- 72
X51861.1|    ----------GTTTTGCCCTTAGACTATTCAGGTTGCCTCTGTGG-----------ATTA--GGGCTGGAGGCAGCACGGATAGTCTGATGGCCA-- 72
L21974.1|    ----------GTTTTGCCCTTAGACTATTCAGGTTGCCTCTGTGG-----------ATTA--GGGCTGGAGGCAGCACGGATAGTCTGATGGCCA-- 72
M21526.1|    ----------GTTTTGCCCTTAGACTATTCAGGTTGCCTCTGTGG-----------ATTA--GGGCTGGAGGCAGCACGGATAGTCTGATGGCCA-- 72
K03523.1|    ----------GTTTTGCCCTTAGACTATTCAAGTTGCCTCTGTGG-----------ATTA--GGGCTGGAGGCAGCACGGATAGTCTGATGGCCA-- 72
M16581.1|    ----------GTTTTGCCCTTAGACTATTCAAGTTGCCTCTGTGG-----------ATTA--GGGCTGGAGGCAGCGCGGATAGTCTGATGGCCA-- 72
AB303223.1|  ----------GTTTCGCCCTTAGACTATTCAAGTTGCCTCTGTGG-----------ATTA--GGGCTGGAGGCAGCTCGGATGGTCTGATGGCCA-- 72
GU982308.1|  ----------GTTTTGCCCCTAGACTATTCAAGTTGCCTCTGTGGGTCTATCGAGAATTA--GGACTGGAGGCAGCTCGGATGGTCTGATGGCCA-- 83
AY689466.1|  ----------GTTTTGCCCCTAGACTATTCAAGTTGCCTCTGTGG-------------------ACAGGAGGCAGCTTGGATGGTCTGATGGCCA-- 66
GU982309.1|  ----------GTTTTGCCCCTAGACTAATCAAGTTGCCTCTGTGG-----------ATTA--GGACTGGAGGCAGCTCGGATGGTCTGATGGCCA-- 72
GU982310.1|  ----------GTTTTGCCCCTAGACTATTCAAGTTGCCTCTGTGG-----------ATTA--GGACTGGAGGCAGCTCGGATGGTCTGATGGCCA-- 78
GU982307.1|  ----------GTTTTGCCCCTAGACTATTCAAGTTGCCTCTGTGG-----------ATTA--GGACTGGAGGCAGCTCGGATGGTCTGATGGCCA-- 81
AF257655.1|  ----------GTTTTGCCCTTAGACTATTCAAGTTGCCTCTGTGG-----------ATTA--GGACTGGAGGCAGCTCAGATGGTCTGATGGCCA-- 72
HM600813.1|  ----------GTTTTGCCCTTAGACTATTCAAGTTGCCTCTGTGG-----------ATTA--GGACTGGAGACAGCTCGGATGGTCTGATGGCCA-- 72
HM600812.1|  ----------GTTTTGCCCTTAGACTATTCAAGTTGCCTCTGTGG-----------ATTA--GGACTGGAGACAGCTCGGATGGTCTGATGGCCA-- 72
HM235668.1|  ----------GTTTTGCCCTTAGACTATTCAAGTTGCCTCTGTGG-----------ATTA--GGACTGGAGGCGGCTCGGATGGTCTGATGGCCA-- 78
HM582657.1|  ----------GTTTTGCCCTTAGACTATTCAAGTTGCCTCTGTGG-----------ATTA--GGACTAGAGGCGGCTCGGATGGTCTGATGGCCA-- 78
AY689454.1|  ----------GTTTTGCCCTTAGACTATTCAAGTTGCCTCTGTGG-----------ATTA--GGACTGGAGGCAGCTCGGATGGTCTGATGGCCA-- 72
AY027920.1|  ----------GTTTTGCCCTTAGACTATTCAAGTTGCCTCTGTGG-----------ATTA--GGACTGGAGGCAGCTCGGATGGTCTGATGGCCA-- 72
AF305091.1|  ----------GTTTTGCCCTTAGACTATTCAAGTTGCCTCTGTGG-----------ATTA--GGACTGGAGGCAGCTCGGATGGTCTGATGGCCA-- 72
AY689444.1|  ----------GTTTGGCCCTTAGACTATTCAAGTTGCCTCTGTGG-----------ATTA--GGACTGGAGGCAGCTCGGATGGTCTGATGGCCA-- 72
AY689449.1|  --------------TGCCCTTAGACTATTCAAGTTGCCTCTGTGG-----------ACTGGTGGACTGGAGGCAGCTCGGATGGTCTGATGGCCA-- 70
AY689441.1|  -------------TTGCCCTTAGACTATTCAAATTGCCTCGGTGG-----------ACTGGTGGACTGGAGGCAGCTCGGATGATCTGATGGCCA-- 71
AY689456.1|  ----------GTTTTGCCCTTAGACTATTCAAGTTGCCTCTGTGG-----------ACTGGTGGACTGAAGGCAGCTTGGATGGTCTGATGGCCA-- 74
AY689455.1|  ----------GTTTTGCCCTTAGACTATTCAAGTTGCCTCTGTGG-----------ACTGGTGGACTGAAGGCAGCTTGGATGGTCTGATGGCCA-- 80
AY689445.1|  ------------TTTGCCCTTAGACTATTCAAGTYGCCTCTGTGG-----------ACTGGTGGACTGAAGGCRGCTCGGATGGTCTGATGGCCA-- 72
AF307949.1|  ----------GTTTTGCCCTTAGACTATTCAAGTTGCCTCTGTGG-----------ACTGGTGGACTGGAGGCAGCTTGGATGGTCTGATGGCCA-- 74
AY689467.1|  -------------TTGCCCTTAGACTATTCAAGTTGCCTCTGTGG-----------ACTGGTGGACTGGAGGCAGCTTGGATGGTCTGATGGCCA-- 71
AY689447.1|  ----------GTTTT-CCCTTAGACTATTCAAGTTGCCTCTGTGG-----------ACTGGTGGACTGGAGGCAGCTTGGATGGTCTGATGGCCA-- 73
AY689460.1|  ----------GTTTTGCCCTTAGACTATTCAAGTTGCCTCTGTGG-----------ACTGGTGGACTGGAGGCAGCTCGGATGGTCTGATGGCCA-- 74
AY689462.1|  ----------GTTTTGCCCTTAGACTATTCAAGTTGCCTCTGCGG-----------ACTGGTGGACTGGAGGCAGCTCGGATGGTCTGATGGCCA-- 74
AY689452.1|  ----------GTTTTGCCCTTAGACTATTCAAGTTGCCTCTATGG-----------ACTGGTGGACTGGAGGCAGCTCGGATGGTCTGATGGCCA-- 74
AY689442.1|  ----------GTTTTGCCCTTAGACTATTCAAGTTGCCTCTA---------------------GACTGGAGGCAGCTCGGATGGTCTGATGGCCA-- 64
EU264064.1|  ----------GTTTCGCCCTTAGACTATTCAAGTTGCCTCTGTTG-----------ACTGATGGACTGGAGGCAGCTCGGATGGTCTGATGGCCA-- 74
FJ216405.1|  ----------GTTTCGCCCTTAGACTATTCAAGCTGCCTCTGTTG-----------ACTGATGGACTGGAGGCAGCTCGGATGGTCTGATGGCCA-- 74
EU264065.1|  ----------GTTTCGCCCTTAGACTATTCAAGTTGCCTCCGTTG-----------ACTGATGGACTGGAGGCAGCTCGGATGGTCTGATGGCCA-- 74
AY689468.1|  ----------GTTTTGCCCTTAGACTATTCAAGTTGCCTCTGTGG-----------ACTGGTGGACTGGAGGCAGCTCGGATAGTCTGATGGCCA-- 74
AY689438.1|  ----------GTTTTGCCCTTAGACTATTCAAGTTGCCTCTGTGG-----------ACTGGTGGACTGGAGGCGGCTCGGATAGTCTGATGGCCA-- 80
AY689450.1|  ----------GTTTTGCCCTTAGACTATTCAAGTTGCCTCTGTGG-----------ACTGGTGGACTGGAGGCAGCTCGGATAGTCTGATGGYCA-- 74
AY689465.1|  ----------GTTTTGCCCTTAGAYTATTCAAGTTGCCTCTG-------------------TGGACTGGAGGCAGCTCGGATGGTCTGATGGCCA-- 66
```

Supplementary Figure S1

Sequence alignment of all E (XSR) pre-miRNA sequences of ALV-J and RSV strains available in the GenBank showing positions of the mature -5p and -3p miRNAs (shaded light blue). The changes in comparison with HPRS-103 sequences are underlined.
